# A Theoretical-Experimental Study on the Structure and Activity of Certain Quinolones and the Interaction of Their Cu(II)-Complexes on a DNA Model

**DOI:** 10.1155/MBD.2000.301

**Published:** 2000

**Authors:** J. Robles, J. Martín-Polo, L. Álvarez-Valtierra, L. Hinojosa, G. Mendoza-Díaz

**Affiliations:** Facultad de Química Universidad de Guanajuato Noria Alta S/n Guanajuato GTO. 36050 Mexico

## Abstract

Theoretical electronic Structure methods have been employed to study the structure and activity of certain (free) quinolones and the interaction of their Cu(II)-complexes on a DNA model (Rhodamine 6G (rhod)). As a manner of assessing the generated geometries, the nalidixic acid geometrical parameters obtained were tested against the crystallographic ones and it was found that the average error in the calculated geometries is small. The present study allows us to (1) Rationalize the observed differences in antibiotic activities through their electronic hardnesses. (2) Suggest a plausible mechanism of action for these drugs through formation of a reactive intermediate (or carrier) which would consist of a quinolone anion coordinated to an adequate metal center (Cu(II) in this study). (3) We find that, through this model of DNA (modeled with rhod) the interaction seems to be mediated by an effective π-π stacking. (4) Finally, an *in vitro* experiment was designed so that the intercalation process in DNA could be experimentally modeled as well. The quenching of the rhod fluorescence is proportional to the strength of the Cu(II)-complex-rhod interaction and therefore provides a quantitative measurement of the “intercalating” capacity of the quinolones and their copper complexes. These results agree well with the theoretical total adduct formation energies.


### A THEORETICAL-EXPERIMENTAL STUDY ON THE STRUCTURE AND ACTIVITY OF CERTAIN QUINOLONES AND THE INTERACTION OF THEIR Cu(II)-COMPLEXES ON A DNA MODEL

J. Robles, J. Martín-Polo, L. Álvarez-Valtierra, L. Hinojosa and G. Mendoza-Díaz*

*Facultad de Química, Universidad de Guanajuato, Noria Alta S/n, Guanajuato, GTO. 36050, México*
*<mendozag@quijote.ugto.mx>*

**Abstract**

Theoretical electronic structure methods have been employed to study the structure and activity of certain (free) quinolones and the interaction of their Cu(II)-complexes on a DNA model (Rhodamine 6G (rhod)). As a manner of assessing the generated geometries, the nalidixic acid geometrical parameters obtained were tested against the crystallographic ones and it was found that the average error in the calculated geometries is small. The present study allows us to (1) Rationalize the observed differences in antibiotic activities through their electronic hardnesses. (2) Suggest a plausible mechanism of action for these drugs through formation of a reactive intermediate (or carrier) which would consist of a quinolone anion coordinated to an adequate metal center (Cu(II) in this study). (3) We find that, through this model of DNA (modeled with rhod ) the interaction seems to be mediated by an effective $\pi-\pi$ stacking. (4) Finally, an *in vitro* experiment was designed so that the intercalation process in DNA could be experimentally modeled as well. The quenching of the rhod fluorescence is proportional to the strength of the Cu(II)-complex-rhod interaction and therefore provides a quantitative measurement of the "intercalating" capacity of the quinolones and their copper complexes. These results agree well with the theoretical total adduct formation energies.

**Key Words**: structure and activity of quinolones, theoretical study, quantum-mechanical calculations, Cu(II)-quinolone complexes, DNA intercalation model.

### Introduction

Quinolone and fluoroquinolone antibiotics are a group of synthetic compounds that have been widely used in therapy against several bacteria [1]. Nalidixic acid was the first compound developed of this series and it has been in use for more than 30 years. Due to its narrow activity spectrum and the development of resistance by several bacteria strains, this compound is now actually used mainly as an antiseptic for the urinary system. Since the first compound was developed, more than 10,000 compounds have been synthesized and several have been introduced into clinical use [2,3]. In **Fig. 1** the structures of some of these compounds (those studied in this paper), are shown. These are: Nalidixic acid (nal), oxolinic acid (oxo), cinoxacine (cnx), norfloxacine (nor), ciprofloxacine (cip), lomefloxacine (lome) and norflocinoxacine (norcin). With exception of the last one, whose name is proposed here, although it was first reported nameless by Matsumoto et al. [4 ], all are commercially available.

All of these compounds act by inhibition of DNA synthesis. However, the interaction mechanism is not completely understood. There is some evidence reported suggesting that these drugs interact directly with the DNA, blocking the activity of the DNA-gyrase [5, 6]. Also, in earlier studies it has been proposed that the action of these drugs may be mediated by a metal ion such as copper or iron [7].

At our laboratory, the mode of coordination of some of these quinolones to copper (II) has been explored for several years [8-10]. It was found that these antibiotics bind to metal ions by the same coordinative groups: 3-carboxylate and 4-oxo. Others, have also found this coordinative behavior in quinolone complexes with other metal ions [11-16]. It was also found that the stability of these complexes is strongly dependent on the metal environment [17,18], i.e., the stability constants depend upon the type of diammine coordinated to the copper ion. All this evidence supports the hypothesis that these drugs may act by mediation of a metal ion.

It was recently reported that the $[Cu(phen)(nal)(H_2O)]^+$ cationic complex, containing phenantroline (phen), acts as a potent chemical nuclease *in vitro* and it also produces damage to DNA *in vivo* [19], being much more effective than the free quinolone or the $[Cu(phen)]^{2+}$ cation, a well known effective chemical nuclease [20].

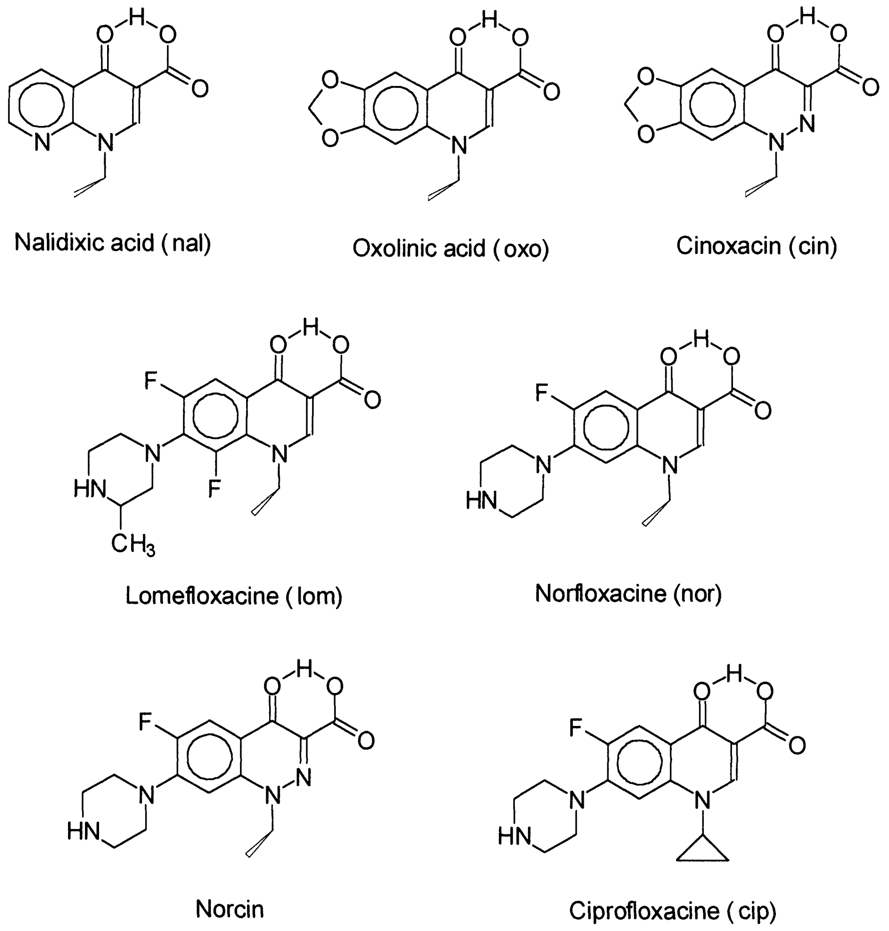

Structures of the 7 free quinolones studied in present work.
**Figure 1**

Considering that the quinolones and their mixed complexes are molecules with high planarity, one may consider that a possible mechanism of action is through their intercalation in DNA. This hypothesis is theoretically modeled and tested in the present paper.

A systematic approach to this hypothesis, is undertaken attempting to rationalize the observed chemical and biological activity of the drugs and their complexes. Quantum mechanical calculations at different levels of theory were performed (depending on the computational complexity of the systems). Results suggest that the DNA intercalation mechanism is plausible. Information is also provided on the structure, activity and electronic properties of:

a) The free antibiotics.
b) Some Cu (II)-complexes formed with these quinolones.
c) The interaction adducts formed through the interaction of these Cu (II)-complexes and a DNA-model.

Theoretical results were compared to an *in vitro* experiment designed so that the intercalation process in DNA could be experimentally modeled as well. The experiments probe into the interaction of the free quinolones or some of their complexes with a fluorophore that mimics the DNA. We chose Rhodamine-6G, (rhod) a well known zwitterionic fluorophore, that may interact with the quinolones or their complexes by a $\pi$–$\pi$ stacking interaction. It is possible to correlate the quenching of the fluorescence with the strength of the interaction and therefore have a quantitative measurement of the "intercalating" capacity of the quinolones and their copper complexes in a DNA-model.

**Materials, equipment and methods:**

*a) Theoretical and Computational Methodology*

Reactivity indexes derived from the Density Functional Theory (DFT) of the electronic structure of molecules [21-22] have been very successfully applied to describe and rationalize chemical reactivity in a very broad range of applications.

Frontier orbitals (highest occupied molecular orbital (HOMO) and lowest unoccupied molecular orbital (LUMO)) of a chemical species are very important to describe its reactivity. This was first recognized by Fukui [23]. Large HOMO-LUMO gaps are known to provide great stability and low reactivity to a chemical species. This observation is a basic ingredient of the *Hard and Soft Acids and Bases* principle of Pearson [24], which was first quantified by means of the DFT [25-27]. Thus, a sound definition was established of the concepts of global (as pertaining to the whole chemical species) or absolute hardness ($\eta$) and softness (S), which are inverse to each other.

Absolute hardness is the second derivative of the ground state electronic energy, (E), of a chemical species with N electrons [25]

$$\eta = \frac{1}{2}\left(\frac{\delta^2 E}{\delta N^2}\right)_{v(r)} \qquad (1)$$

where v(**r**) is the potential imposed at position **r** by the nuclei. A finite difference approximation [21,25] yields

$$\eta = \frac{I - A}{2} \qquad (2)$$

where I is the vertical ionization potential and A the electron affinity. Furthermore, in the molecular orbital theories where the Koopmans Theorem is valid [21],

$$\eta = \frac{1}{2}(\varepsilon_{LUMO} - \varepsilon_{HOMO}) \qquad (3)$$

where $\varepsilon_{LUMO}$ and $\varepsilon_{HOMO}$ are the LUMO and HOMO orbital energies of the species, respectively. Therefore, hardness is proportional to the HOMO-LUMO gap and large $\eta$ implies stability for the chemical species. Absolute or global softness is [21,28-32],

$$S = \left(\frac{1}{2\eta}\right) = \left(\frac{\delta N}{\delta \mu}\right)_{v(r)} \qquad (4)$$

where $\mu$ is the electronic chemical potential [21] of the species. Using again a finite difference approximation (assuming energy depends quadratically with N),

$$S \approx \left(\frac{1}{I - A}\right) \qquad (5)$$

Atomic and molecular global hardnesses have been computed by different means [33-36] and the concept of hardness has been used to understand aromaticity in organic [37-38] and organometallic molecules [39]. The *Relative* hardness concept was introduced to characterize aromatic and anti-aromatic species [40] and the activation hardness index is useful to predict the orientation of electrophilic aromatic substitution in organic chemistry [41]. A very important result is the recognition that there seems to be a rule of nature requiring that *molecules arrange themselves so as to be as hard as possible* [42]. This has been rationalized through the principle of maximum hardness, which indicates that the equilibrium state of a chemical system is attained when $\eta$ is maximum (minimum S) [30,43-45].

*b) Computers and software employed.*

All calculations (Semiempirical PM3, *Ab Initio* Hartree- Fock (HF) and Density Functional Theory (DFT) ) were carried out on a Silicon Graphics Octane Workstation (Dual MIPS RISC R10000, 64-bit, 195 MHz/1MB cache Processors, IRIX 6.4 operating system, 256 MB RAM, 4.0 GB Disk).

For the present calculations and visualizations, the Gaussian 94 [46] and the Spartan V5.1 software packages were employed [47].

*c) Sample preparation and Fluorescence Experiments:*

Fluorescence measurements were made in a Perkin-Elmer LS-5B luminiscence spectrometer, using 1 mL fluorescence disposable cuvettes with a 275 nm cutoff, with a Tris/HCl pH 7.95 buffer as blank. Excitation wavelength number was 301 nm and emission was read at 548 nm.

The complexes were prepared accordingly with the methods previously reported [8,9]. Nalidixic acid, cinoxacine, 1,10-phenanthroline and 2,2'-bipyridine were purchased from Sigma-Aldrich Química S. A. De C. V.

Sample preparation for fluorescence measurements was carried out as follows: 10 μL of $10^{-4}$ M Rhod in Tris/HCl buffer pH 7.95 (pH and ionic strength were kept for every sample) were placed in 1.5 mL *ependorff* tubes. These and each compound under test were taken up to 1000 μL, using the same buffer. A $10^{-3}$ M solution of the antibiotics and complexes to be tested were added as follows to each ependorff tube: 0,10, 15, 20, 25, 30, 35, 40, 45, 50 and 55 μL. This corresponds to a final concentration of: 0, 1.5, 2.0, 2.5, 3.0, 3.75, 4.5, 5.25, 6.0, 6.75, 7.5 and 8.25 X $10^{-5}$ M.

### Results and Discussion

*a) Free Quinolones.*

For each chemical species, we initially obtained a Semi-empirical [48-49] PM3 geometry, which in turn was used as initial geometry to obtain a Hartree-Fock minimum basis set (RHF/STO-3G) optimized geometry [48]. The obtained geometry was tested to correspond to an absolute potential minimum through analysis of the resulting Hessian frequencies. Keeping this geometry, we thereafter obtained single point energies and orbital energies at a higher level of theory [48], improving the numerical basis set, namely to RHF/3-21G(d). Therefore the achieved level of theory for free quinolones is RHF/3-21G(d)// RHF/STO-3G.

Since there are no available experimental geometries for some of these quinolones, we test the optimized geometries, comparing the theoretical geometrical parameters for Nalidixic acid with the experimental ones [50]. It was found that for this molecule, in the average, the present method overestimates the experimental bond lengths by 3.06%, while the bond angles are underestimated by 4.11%. It is assumed that the other quinolones would yield comparable errors, which are acceptable for the purposes of this work.

Results (at this level of theory) for a number of properties (hardness (η), dipole moment (μ), ionization potential (I), electron affinity (A), electronegativity (χ)),of the 7 free quinolones, together with the hidrophobicity in vitro [1] and the minimal inhibitory concentration (MIC) [1] are displayed in **Table 1.**

**Table 1. Results for the 7 free Quinolones. Theory level is RHF/3-21G(*)//RHF/STO-3G.**

| *Quinolone* | *Dipole (Debyes)* | *I (eV)* | *A (eV)* | *χ (eV)* | *η (eV)* | *S ($eV^{-1}$)* | *Hidrophobicity in vitro* | *MIC* (mg/ml) |
|---|---|---|---|---|---|---|---|---|
| norcin | 10.65 | 8.99 | -0.97 | 4.01 | 9.96 | 0.1004 | -- | 3.13 |
| cnx | 10.39 | 9.09 | -0.96 | 4.07 | 10.05 | 0.0995 | 0.03 | 3.10 |
| nor | 10.64 | 8.77 | -1.71 | 3.53 | 10.48 | 0.0954 | 0.01 | 0.125 |
| lome | 10.14 | 9.00 | -1.57 | 3.72 | 10.57 | 0.0946 | -- | 0.12 |
| oxo | 10.30 | 8.87 | -1.70 | 3.59 | 10.57 | 0.0946 | 2.23 | 0.20 |
| cip | 11.52 | 8.93 | -1.71 | 3.61 | 10.64 | 0.0940 | 0.02 | 0.06 |
| nal | 9.95 | 9.23 | -1.58 | 3.83 | 10.81 | 0.0925 | 8.92 | 6.30 |

In **Figure 2 (a)** a plot of η (HOMO-LUMO gap), vs. the logarithm of MIC of the free quinolones is shown. A negative linear tendency is observed indicating that:

i) The greater the hardness the smaller the MIC, predicting higher antibiotic activity. Such tendency contradicts the expected fact that the softer species should be the most reactive.
ii) However, it is observed that this plot groups the antibiotics depending on their aromatic system: a formal quinolone, a naphtiridone or a cinnolone.

A plot of χ versus the logarithm of MIC is displayed in **Figure 2 (b).** A tendency where the most active compounds are those with the lower electronegativity is observed. This seems to indicate that the most active free quinolones are those with the highest ability to donate electrons, as measured by their low electronegativities. This is important for effective π-π charge transfer. Again, it is possible to classify the drugs in terms of their parent ring system.

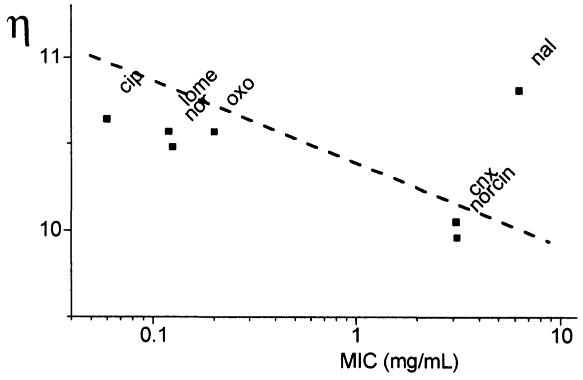

**2 (a)**

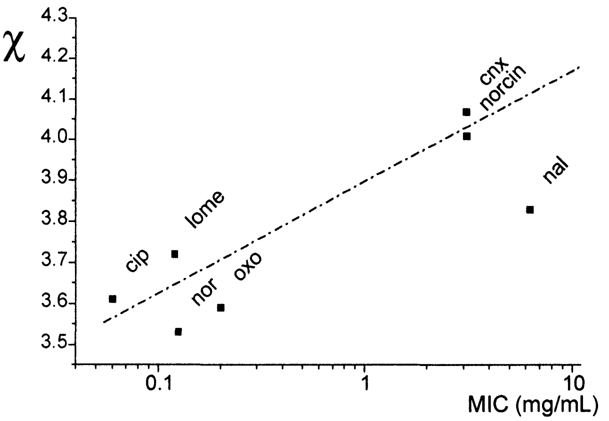

**2 (b)**

**(a)** Hardness *vs*. MIC for 7 free quinolones, **(b)** Electronegativity *vs*. MIC for the 7 free quinolones.

**Figure 2**

*b) Cu- complexes.*

For each Cu- complex, due to its computational difficulties, we employed an optimized SYBYL molecular mechanics geometry. Since the Cu atom parameters are not very reliable in SYBYL, they were set to the Cu-atom experimental parameters [8] in $[Cu(phen)(nal)(H_2O)]^+$ as restrictions during the otherwise full optimization. This constraint allows one to reproduce the observed square pyramid geometry around the Cu ion. We thereafter obtained single point energies and orbital energies at a higher level of theory [48], namely to the Kohn and Sham DFT functional of Vosko, Wilkes and Namur (VWN), within the local density approximation and with the DN(*) numerical basis set (equivalent in size to a 6-31G(d) Gaussian basis set [21,47]. Therefore, the achieved level of theory for Cu-complexes calculations is DFT-VWN/DN(*)// SYBYL.

Total formation enthalpies for 3 complexes have been computed, namely $[Cu(bipy)(cnx)(H_2O)]^+$, $[Cu(phen)(nal)(H_2O)]^+$ and $[Cu(phen)(cip)(H_2O)]^+$ at this level of theory. The obtained geometries are shown in **Figure 3**. Formation enthalpy of each complex is computed by subtracting from the complex the energies of its components. Results are shown in **Table 2**. The most stable complex is predicted to be $[Cu(bipy)(cnx)(H_2O)]^+$ and stabilities apparently are similar in the 3 complexes. This is in agreement with the experimental stability constants reported for similar complexes [18]. Since energy values are quite large, this may suggest that these complexes could remain stable *in vivo* and the observed antibiotic action would be due to the entire complex rather than an eventual segregation of the parts and independent action of them. These results support the idea of a synergic rather than additive action.

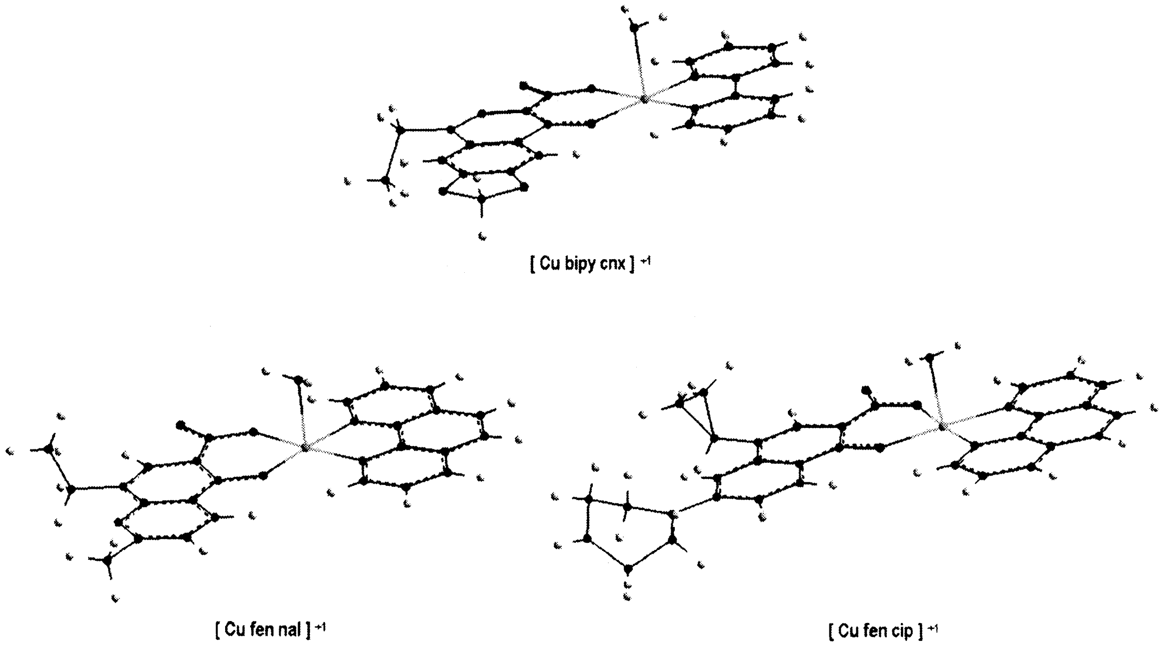

Optimized geometries for the three complexes: $[Cu(bipy)(cnx)(H_2O)]^{+1}$, $[Cu(phen)(nal)(H_2O)]^{+1}$ and $[Cu(phen)(cip)(H_2O)]^{+1}$.

**Figure 3**

Although in the previous section we discussed the ability of the free quinolones to perform as π-π electron transfer species, as measured from the free quinolones electronegativities, now we can observe how their hardnesses may be modified by formation of the Cu-complexes where the quinolones participate as coordination ligands. The Shen hypothesis [5, 6] suggests the direct interaction of the free quinolones with the DNA double helix but it has also been proposed that this interaction could be assisted by a metal ion like Cu or Fe [6]. An alternated stacking between the cnx and phen, [9] has been observed, displaying intermolecular distances similar to those in the DNA double helix. Thus, these complexes could indeed be good intercalating species within the DNA. This is probed through calculation of the Cu-complexes hardnesses. In **Figure 4**, $\eta$ vs. MIC for the complexes are plotted. The situation changes dramatically from the trend observed in Figure 2. Now the SOFTER complexes correspond to the ones whose quinolone has the largest reported MIC value. This result taken together with the results for the free quinolones suggest that the antibiotics must be associated with the "metal carrier" before they can perform with the observed activity inside the cell.

**Table 2. Cu-complexes formation enthalpies. Theory level is DFT-VWN/DN(*)// SYBYL.**

| Complex | Formation enthalpy (Hartrees) |
|---|---|
| $[Cu(bipy)(cnx)(H_2O)]^+$ | -76.4937 |
| $[Cu(phen)(nal)(H_2O)]^+$ | -76.4151 |
| $[Cu(phen)(cip)(H_2O)]^+$ | -76.2722 |

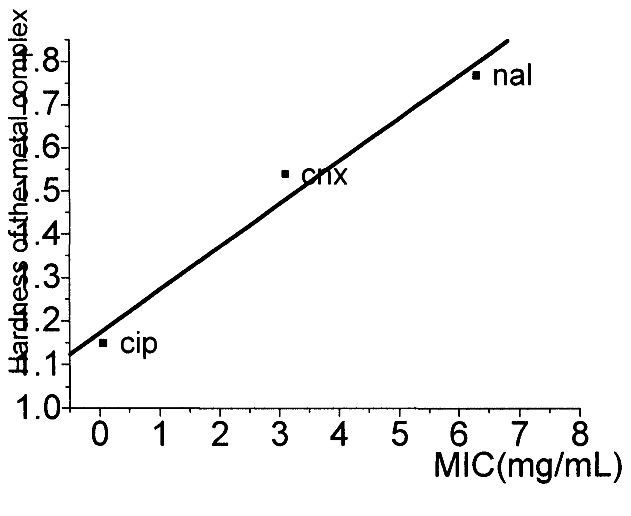

MIC of the free quinolones *vs*. hardness for the 3 Cu(II)- complexes studied.

**Figure 4**

In **Table 3**, $\chi$ and other electronic properties (I, A and $\eta$) of these systems are shown. In open shell systems, (when the Cu ion is present), the formulae derived for these properties by Vargas and Vela [51] have been employed,

$$I = \frac{1}{2}\left[\varepsilon_{L\beta} - 2\varepsilon_{H\alpha} - \varepsilon_{H\beta}\right] \tag{6}$$

$$A = \frac{1}{2}\left[\varepsilon_{H\alpha} - 2\varepsilon_{L\beta} - \varepsilon_{L\alpha}\right] \tag{7}$$

$$\eta = \frac{1}{2}\left[\varepsilon_{L\alpha} - \varepsilon_{H\beta}\right] - \frac{3}{2}\left[\varepsilon_{H\alpha} - \varepsilon_{L\beta}\right] \tag{8}$$

$$\mu = -\chi = \frac{1}{4}\left[\varepsilon_{L\alpha} + \varepsilon_{L\beta} + \varepsilon_{H\alpha} + \varepsilon_{H\beta}\right] \tag{9}$$

where the $\alpha$ and $\beta$ indexes label the two different HOMO (H) and LUMO (L) spinorbitals obtained in these spin-polarized calculations.

From these data, it is possible to observe that the less electronegative system is the most active drug which is also the softer, as expected. In this case, it is contrary to the free quinolones behavior shown in Table 1. The complex with cip displays the lowest hardness; predicted to be the most active species, as observed.

*Cu- complexes- Rhodamine 6G adducts.*

For each adduct, Cu-complex- Rhod, due to the computational difficulties, we employed an optimized SYBYL molecular mechanics geometry on the best conformer obtained from a systematic *SYBYL conformer analysis*. The Cu atom SYBYL parameters again were set to the Cu-atom experimental parameters in $[Cu(phen)(nal)(H_2O)]^+$ as restrictions during the otherwise full optimization. Also, the rhod molecule was slightly simplified to reduce the computational cost by modeling the 3 ethyl groups by methyl ones. Keeping this geometry, we thereafter obtained single point energies and orbital energies at the Semiempirical PM3(tm) level (parameters include d-orbitals for Cu) [47-49]. Therefore the achieved level of theory for the Cu- complex-rhod adducts is PM3(tm)// SYBYL.

**Table 3. Electronic properties of rhod, some Cu-complexes and their adducts (complex-rhod). Theory level is DFT-VWN/DN(*)// SYBYL for the complexes and PM3(tm)// SYBYL for the adducts..**

| Species | I (eV) | A (eV) | $\chi$ (eV) | $\eta$(eV) |
|---|---|---|---|---|
| $rhod^+$ | 8.43 | 7.12 | 7.68 | 1.32 |
| $[Cu(bipy)(cnx)(H_2O)]^+$ | 7.95 | 6.41 | 7.18 | 1.54 |
| $[Cu(phen)(nal)(H_2O)]^+$ | 8.24 | 6.47 | 7.35 | 1.77 |
| $[Cu(phen)(cip)(H_2O)]^+$ | 7.12 | 5.97 | 6.55 | 1.15 |
| rhod-$[Cu(bipy)(cnx)(H_2O)]^+$ | 16.53 | 5.18 | 10.85 | 11.36 |
| rhod-$[Cu(phen)(nal)(H_2O)]^+$ | 17.05 | 4.89 | 10.97 | 12.16 |
| rhod-$[Cu(phen)(cip)(H_2O)]^+$ | 14.40 | 5.56 | 9.98 | 8.84 |

The geometries of the adducts obtained through this procedure seem to be physically and chemically reasonable. In **Table 4**, The calculated average intercalation distance within each adduct is displayed They are all quite close to the typical x-ray measured distances at approximately 3.4 Å found in DNA double helix.

In the lower half of **Table 3**, $\chi$ and other electronic properties, I, A, and $\eta$ of these systems are shown computed at this level of theory. In the open shell systems, the formulae derived by Vargas [51] have been employed again.

We have computed total formation enthalpies for 3 adducts, namely rhod-$[Cu(bipy)(cnx)(H_2O)]^+$, rhod-$[Cu(phen)(nal)(H_2O)]^+$ and rhod-$[Cu(phen)(cip)(H_2O)]^+$. Results are shown in **Table 5**. The latter adduct is predicted to be the most stable one.

**Table 4. Intercalation distances calculated for each adduct. Theory level is PM3(tm)// SYBYL**

| Adduct | Intercalation distance (Å) |
|---|---|
| rhod-$[Cu(phen)(nal)(H_2O)]^+$ | 3.18 |
| rhod-$[Cu(bipy)(cnx)(H_2O)]^+$ | 3.35 |
| rhod-$[Cu(phen)(cip)(H_2O)]^+$ | 3.19 |

**Table 5. Quenching of fluorescence and formation enthalpies of the Cu-complex-rhod adducts. Theory level is PM3(tm) // SYBYL.**

| Adduct (with rhod) | Formation enthalpy (eV) | $K_{SV}$ | log(-$K_{SV}$) |
|---|---|---|---|
| $[Cu(phen)(Nal)(H_2O)]$ | -10707.69 | 16539 | 4.218 |
| $[Cu(bipy)(Cnx)\ )(H_2O)]$ | -11024.98 | 27689 | 4.442 |
| $[Cu(phen)(Cip)\ )(H_2O)]$ | -11993.07 | 20389 | 4.310 |

In **Fig. 5**, frontier orbitals maps (a) of the complex $[Cu(phen)(nal)(H_2O)]^+$, (b) Rhodamine 6G and (c) the adduct rhod-$[Cu(phen)(nal)(H_2O)]^+$ are displayed. These maps are similar to the other adducts so only this one is presented. In all cases the larger contribution to the adduct's HOMO stems from the Copper-complex HOMO, apparently always being the charge donor. The largest contribution to the adduct's LUMO comes from the rhod's LUMO, thus behaving as the $\pi$-charge acceptor. The MO space distribution indicates the feasibility of the $\pi$-$\pi$-electron transfer. Energetically this is also favored due to the vicinity between the corresponding frontier-orbitals energies, HOMO in the complex at -0.2932 a.u. and LUMO in Rhod at -0.2617 a.u.

Furthermore, the molecular electronegativity of rhod is larger than the value of the complex, (**Table 3**). This parameter is indicative of the ability of rhod to withdraw $\pi$-electrons toward itself from the complex.

From these data, it seems feasible that in the mechanism of action of these drugs, one important step should be the intercalation of the quinolone complexed to a metal center. The theoretical calculations and the experimental model (*vide infra*) follow the observed activity of the drugs.

*c) Fluorescence quenching experiments*

Since the discovery of metallo-intercalators into DNA, several groups have studied the potential activity of these species as antineoplasic compounds. In order to screen the complexes that could be useful in cancer therapy one possible procedure is to measure the intercalating ability of these metal-complexes. One approach to measure this intercalating ability is to study the interaction of the metal-complexes with a fluorophore, in this case (rhod). The quenching of the observed fluorescence in the hydrophobic fluorophore (rhod) is proportional to the strength of the interaction [52].

The fluorescence quenching provides a quantitative measurement of the interaction. The Stern-Volmer equation [52] describes the collisional quenching of the fluorescence

$$F_o/F = k_q\tau_o[Q] = K_{SV}\,[Q]$$

Where $F_o$ and F are the intensities of the fluorescence in absence and presence of the quencher, respectively, $k_q$ is the bimolecular constant of quenching, $\tau_o$ is the half-life of the fluorophore in absence of the quencher and [Q] is the concentration of the quencher. It is possible to obtain a global constant, the so-called Stern-Volmer constant, $K_{SV} = k_q\tau_o$. Normally, the quenching data are represented in a $F_o/F$ vs [Q] plot, with a slope equal to $K_{SV}$.

Therefore, we suggest that the most stable adduct (stronger interaction) should have the most negative formation enthalpy and the smallest measured fluorescence (largest abatement), i.e. the larger $K_{SV}$ value. Results are displayed in **Table 5.** When we compare complexes with the same diammine ligand, it is found some correspondence between the theoretical and the experimental values. When phen is the diammine ligand, the best interaction turns out to be between the $[Cu(phen)(cip)(H_2O)]^+$ complex and rhod. We find the least favorable interaction, between the $[Cu(phen)(nal)(H_2O)]^+$ complex and rhod. For these two adducts (both containing phen), there is full agreement between experiment and calculation. The complex with bipy behaves experimentally as the most efficient quencher. This may be explained in term of the efficiency in the energy transfer between the complex and rhod, and could be strongly dependent on the diammine (bipy) planarity and number of rings.

Finally, it is also important to consider that biological activity involves other parameters that are not considered here such as the difference in permeability throughout membranes. However, our results suggest that these drugs may interact inside the cell with a metal center, producing a strong electronic change in the molecule. Our results show that this may reduce the free antibiotic hardness and therefore increase their reactivity in order to display the observed biological activity and that this may involve an intercalation process.

**Acknowledgements**

We gratefully acknowledge financial support from CONACYT through Projects Number 25059-E and 32070-E and from PROSAA (*Programa de Superación Académica y Administrativa-* University of Guanajuato). JR is grateful to DGSCA-UNAM for providing a supercomputer account. L.H. and L.A are thankful to the University of Guanajuato for a student grant during the *IV Verano de la Investigación Científica.*

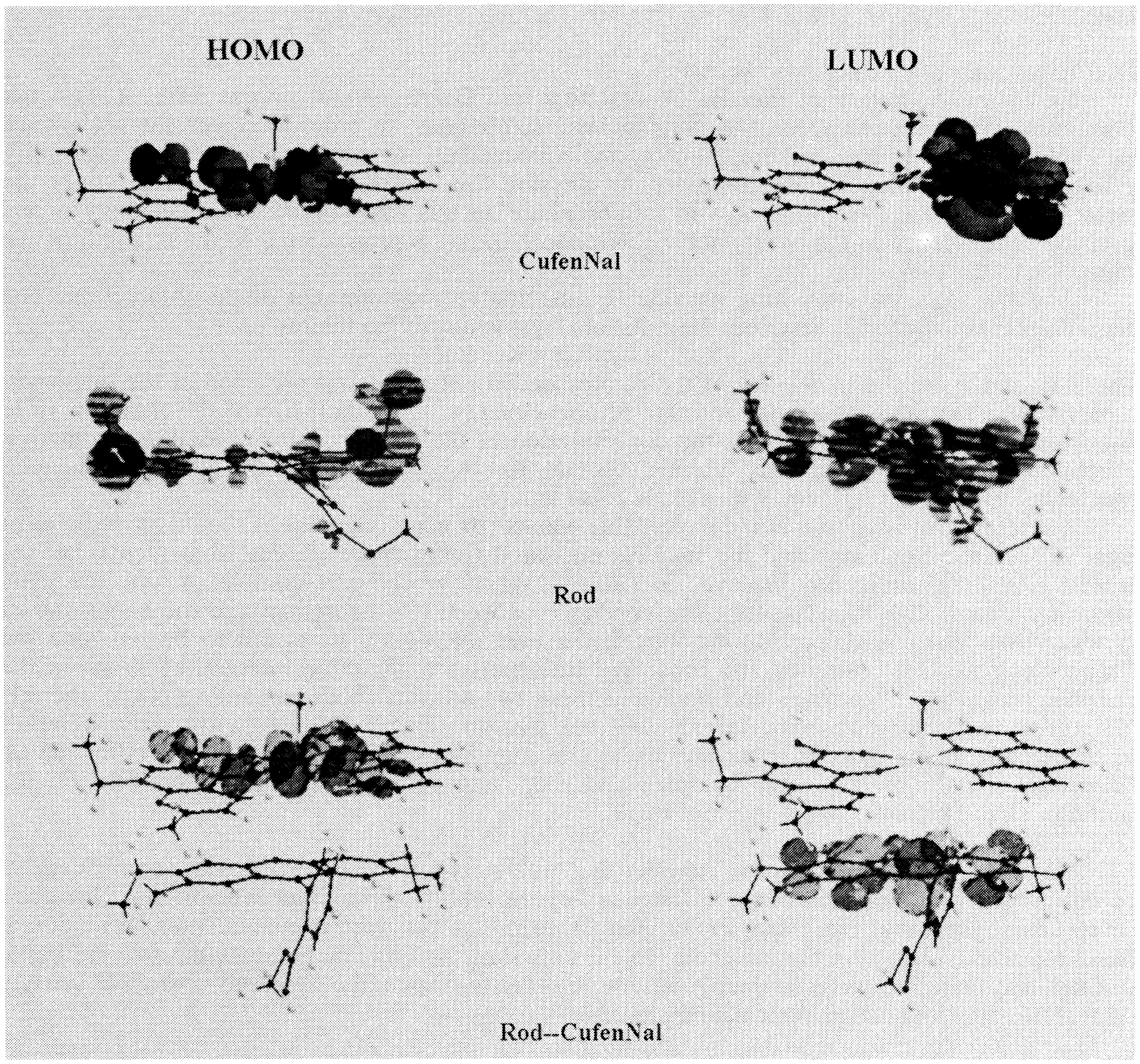

Frontier orbitals maps (a) of the complex $[Cu(phen)(nal)(H_2O)]^+$, (b) of Rhodamine 6G and (c) of the adduct rhod- $[Cu(phen)(nal)(H_2O)]^+$. All HOMOS are in left side and LUMOS in right side.

**Figure 5**

**REFERENCES:**

1. Hooper D. C. and Wolfson J. S., (eds), (1993) *Quinolone Antimicrobial Agents*, , Second Edition, American Society for Microbiology, Washington, D. C., pp.: 1-2
2. Mitscher L. A., Devasthale P. and Zavod R.; Structure-Activity Relationships in Hooper D. C. And Wolfson J. S., Editors, *Quinolone Antimicrobial Agents*, (1993), Second Edition, American Society for Microbiology, Washington, D. C., pp.: 3-49
3. Grohe K., (1992), *Chem. In Brit.*, 34-36
4. Matsumoto J., Miyamoto T.; (1989). Cinoxacin analogues as potential antibacterial agents: synthesis and antibacterial activity. In P. B. Fernandes (ed.), *International Telesymposium on Quinolones*. M. Prious Science Publishers, Barcelona, Spain, p 109-118
5. Shen L. L. and Pernet A. G., (1985), *Proc. Natl. Acad. Sci., USA*, **82**: 307-311
6. Shen L. L., Quinolone-DNA Interaction in Hooper D. C. And Wolfson J. S., Editors, *Quinolone Antimicrobial Agents* (1993), Second Edition, American Society for Microbiology, Washington, D. C., pp 77-95
7. Timmers K. and Sternglanz R., (1978), *Bioinorg. Chem.*, **9**: 145-155
8. Mendoza-Diaz G., Martinez-Aguilera L. M. R., Perez-Alonso R., Solans X. and Moreno-Esparza R. (1987) *Inorg. Chim. Acta*, **138**: 41-47

9. Mendoza-Díaz G., Martinez-Aguilera L. M. R., Moreno-Esparza R., Pannell K. H. and Cervantes-Lee F. (1993) *J. Inorg. Biochem.* **50**: 65-78
10. Mendoza-Díaz G. and Ireta-Moreno J. (1994) *J. Inorg. Biochem.***54**: 235-246
11. Chulvi C., Muñoz M. C., Perelló L., Ortiz R., Arriortua M. I., Via J., Urtiaga K., Amigó J. M. And Ochando L. E. (1991) *J. Inorg. Biochem.* **42**: 133-138
12. Ruiz M., Ortiz R., Perello L., Castiñeiras A., Quirós M., (1993), *Inorg. Chim. Acta,* **211**: 133-139.
13. Riley C. M., Ross D. L., Vander Velde D. And Takusagawa F., (1993), *J. Pharm. & Biomed. Analysis,* **11**: 49-59.
14. Ruiz M., Ortiz R., Perello L., García-Granda S. and Díaz M. R., (1994), *Inorg. Chim. Acta*, **217**:149-154.
15. Turel I., Leban I. and Bukovec N., (1994), *J.Inorg. Biochem.* **56**: 273-282.
16. Ruiz M., Perello L., Ortiz R., Castiñeiras A., Maichle-Mössmer C. and Cantón E., (1995), *J.Inorg. Biochem.* , **59**: 801-810.
17. Wallis S. C., Gahan L. R., Charles B. G., Hambley T. W. And Duckworth P. A., (1996), *J.Inorg. Biochem.* **62**: 1-16
18. Mendoza-Díaz G., Perez-Alonso R. and Moreno-Esparza R. (1996) *J. Inorg. Biochem.* **64**: 207-214
19. Ramírez-Ramírez N., Mendoza-Díaz G., Gutiérrez-Corona F., Pedraza-Reyes M, (1998) , *J. Biol.Inorg. Chem.* **3**: 188-194.
20. Sigman D. S., Mazmunder A. And Perrin D. M., *Chem. Rev.*, **93**: 2295-2316.
21. R. G. Parr and W. Yang (1989), *Density Functional Theory of Atoms and Molecules*, Oxford University Press, New York.
22. B. G. Baekelandt, A. Cedillo and R. G. Parr, (1995) *J. Chem. Phys.* **103**: 8548.
23. K. Fukui, T. Yonezawa and H. Shingu, (1952), *J. Chem. Phys.* **20**: 722.
24. R.G. Pearson, (1973), *Hard and Soft Acids and Bases*, Dowden, Hutchinson and Ross, Stroudsburgs, PA.
25. R. G. Parr and R. G. Pearson, (1983), *J. Am Chem. Soc.* **105**: 7512
26. .R. F. Nalewajski . (1984) *J. Am. Chem. Soc* **106**: 944.
27. P. K. Chattaraj, H. Lee and R. G. Parr (1991) *J. Am. Chem. Soc.* **113**: 1855.
28. M. Berkowitz and R. G. Parr (1988) *J. Chem. Phys.* **88**: 2554.
29. J. L. Gázquez, A. Vela and M. Galván (1987) *Structure and Bonding* **66**: 80.
30. J. L. Gázquez (1993) *Structure and Bonding* **80**: 28.
31. P. K. Chattaraj, A. Cedillo and R.G. Parr (1995) *J. Chem. Phys.* **103**: 10621.
32. R. Balawender and L. Komorowski (1998) *J. Chem. Phys* **109**: 5203.
33. J. Robles and L.J. Bartolotti, (1984) *J. Am. Chem. Soc.* **106**: 3723.
34. L. Baumer, G. Sala and G. Sello (1990) *J. Comp. Chem.* **11**: 694.
35. S. Liu, F. De Proft and R.G. Parr (1997) *J. Phys. Chem. A* **101**: 6991.
36. M. K. Harbola, R. G. Parr and C. Lee (1991) *J. Chem. Phys.* **94**: 6055.
37. Z. Zhou, R.G. Parr and J. F. Garst (1988) *Tetrahedron Lett.* **29**: 4843.
38. Z. Zhou and H. V. Navangul (1990) *J. Phys. Org. Chem.* **3**: 784.
39. J. A. Chamizo, J. Morgado and P. Sosa (1993) *Organometallics* **12**: 5005.
40. Z. Zhou and R.G. Parr (1989) *J. Am. Chem. Soc.* **111**: 7371.
41. Z. Zhou and R. G. Parr (1990) *J. Am. Chem. Soc.* **112**: 5720.
42. R. G. Pearson (1987) *J. Chem. Educ.* **64**: 561.
43. R. G. Parr and P. K. Chattaraj (1991) *J. Am. Chem. Soc.* **113**: 1854.
44. R. G. Pearson (1994) *J. Phys. Chem.* **98**: 1989.
45. J. L. Gázquez, A. Martínez and F. Méndez. (1993) *J. Phys. Chem.* **97**: 4059.
46. Gaussian 94, Revision E.2, M. J. Frisch, G. W. Trucks, H. B. Schlegel, P. M. W. Gill, B. G. Johnson, M. A. Robb, J. R. Cheeseman, T. Keith, G. A. Petersson, J. A. Montgomery, K. Raghavachari, M. A. Al-Laham, V. G. Zakrzewski, J. V. Ortiz, J. B. Foresman, J. Cioslowski, B. B. Stefanov, A. Nanayakkara, M. Challacombe, C. Y. Peng, P. Y. Ayala, W. Chen, M. W. Wong, J. L. Andres, E. S. Replogle, R. Gomperts, R. L. Martin, D. J. Fox, J. S. Binkley, D. J. Defrees, J. Baker, J. P. Stewart, M. Head-Gordon, C. Gonzalez, and J. A. Pople, (1995) Gaussian, Inc., Pittsburgh PA,.
47. *Spartan version 5.1*, (1998), Wavefunction, Inc., 18401 Von Karman Ave., Suite 370, Irvine, CA.
48. Hehre W. J., Radom L., Schleyer P. V. R. and Pople J. A. (1986) *Ab Initio Molecular Orbital Theory*, Wiley, New York.
49. Stewart, JJP. (1989) J.Comp. Chem. **10**: 209
50. Huber C: P., Sake-Gowda D. S: and Acharya K. R. (1980) *Acta Cryst.*, **B36**: 497-499.
51. R. Vargas.(1997), Ph.D. Dissertation, Universidad Autónoma Metropolitana-Iztapalapa, México
52. MacDonald R. I. (1990) Journal of Biological Chemistry, **264**:13533-13539

**Received: January 11, 2001 - Accepted: January 21, 2001 - Received in revised camera-ready format: January 22, 2001**

